# The frequency of follicle stimulating hormone receptor gene polymorphisms in Iranian infertile men with azoospermia

**Published:** 2015-11

**Authors:** Behrouz Gharesi-Fard, Zahra Ghasemi, Saeed Shakeri, Shabnam Behdin, Fatemeh Aghaei, Zahra Malek-Hosseini

**Affiliations:** 1 *Infertility Research Center, Shiraz University of Medical Sciences, Shiraz, Iran.*; 2 *Department of Immunology, School of Medicine, Shiraz University of Medical Sciences, Shiraz, Iran.*; 3 *Proteomics Laboratory, School of Advanced Medical Sciences and Technologies, Shiraz, Iran.*; 4 *Islamic Azad University, Fars Science and Research Branch, Shiraz, Iran.*; 5 *Student Research Center, Shiraz University of Medical Sciences, Shiraz, Iran.*

**Keywords:** *FSH receptor*, *Male infertility*, *Polymorphism*, *Azoospermia*

## Abstract

**Background:**

Azoospermia is the medical condition of a man not having any measurable level of sperm in his semen. Follicle stimulating hormone (FSH) is a member of the glycoprotein hormone family that plays an important role in human reproduction because of its essential role in normal spermatogenesis. Various Single Nucleotide Polymorphisms (SNPs) have been reported within FSH receptor (FSHR) gene that may affect the receptor function.

**Objective:**

The present study aimed to investigate the correlation between two FSHR SNPs at positions A919G, A2039G, and susceptibility to azoospermia in a group of Iranian azoospermic men. The association between FSH levels within the sera and A919G and A2039G alleles and genotypes were also investigated.

**Materials and Methods:**

This case control study was performed on 212 men with azoospermia (126 non-obstructive and 86 obstructive) and 200 healthy Iranian men. Two FSHR gene SNPs were genotyped using PCR-RFLP method. The relationship between FSH levels within the sera and A919G and A2039G alleles and genotypes were also investigated.

**Results:**

Statistical analysis indicated that at A919G position, AA genotype and A allele were more frequent in obstructive azoospermia cases compared to non-obstructive or normal men (p=0.001). Regarding A2039G polymorphisms, no significant difference was observed between both azoospermia groups and the controls. The mean level of serum FSH was higher in the non-obstructive men compared to the obstructive patients (23.8 versus 13.8, respectively, p= 0.04).

**Conclusion:**

The results of the present study indicated that the genetic polymorphisms in the FSHR gene might increase the susceptibility to azoospermia in Iranian men.

## Introduction

The number of couples which meet the definition of infertility at reproductive ages is increasing worldwide. Infertility has different causes and conditions, which may root from congenital disorders or morbidity in males/females (1). One of the most known conditions of infertility in male individuals is azoospermia, defined as the complete absence of spermatozoa in the semen (2). Azoospermia does not mean complete sterility, because many azoospermic men may have varying levels of sperm within their testes. Azoospermia presents in two forms, named obsteructive azoospermia (OA) and non-obstructive azoospermia (NOA). While in OA form the spermatogenesis is normal, due to the absence of vasa deferentia or a mechanical blockage sperm cells are not present in the ejaculatory fluid (3). In contrast, in NOA form spermatogenesis is impaired (2). Several factors may predispose men to the OA or NOA syndromes, including congenital abnormalities, infection, medication, trauma, prostatic cysts, surgical procedure, and endocrine disorders (2, 4). Follicle stimulating hormone (FSH) is essential for normal reproductive function in males and females. FSH is fundamental for Sertoli and granulosa cells function and also for a normal gametogenesis. In the male, FSH is generally considered essential for the pubertal initiation of spermatogenesis and maintenance of quantitative normal sperm production in adults (5). FSH acts through its specific receptor named FSHR. FSHR as a member of G protein-coupled receptor family, after binding to its ligand outside the cell, promotes a signal transduction pathway within the target cell that finally leads to cellular response (6). FSH acts through the FSHR, which is expressed only in Sertoli cells in humans. The FSHR gene consists of 10 exons and 9 introns (7). Mutation screening of the FSHR gene revealed various single nucleotide polymorphisms (SNPs) both in the core promoter and in the coding region, which may affect receptor function (8). Among several SNPs within the FSHR gene, A919G and A2039G have been reported that may affect the receptor function (8).

There are several studies regarding FSHR SNPs and its relationship with infertility. However, there are only a few publications regarding the relationship between FSHR SNP and azoospermia. 

Thus, the present study aimed to investigate the correlation between FSHR gene polymorphism (A919G and A2039G) and susceptibility to azoospermia in a group of Iranian infertile men from Fars province (Iran) in comparison to a healthy control group. The association between FSH levels within the sera and A919G and A2039G alleles and genotypes were also investigated.

## Materials and methods

**Subjects**

Due to the ethical concern of the research, all the participants were ascertained that their blood samples would be used for research purposes and a written consent were obtained from them. In addition, the study was approved by the ethics committee, Shiraz University of Medical Sciences.

This case control study was performed on 212 primary azoospermic patients (126 non-obstructive, mean age 36.2 ± 3.7 years, and 86 obstructive, mean age 35.4 ± 4.2 years) and 200 healthy men (mean age 35.3 ±4. 6 years) who were referred to the Infertility Research Center, Mother and Child Hospital, Shiraz University of Medical Sciences between May 2011 and May 2013. The cases and controls were selected based on age and ethnic was matched from the same geographic region (Fars province, southwest of Iran). The clinical condition of the patients and the type of azoospermia were obtained from their medical records, checked and confirmed by a urologist. At first, azoospermia was confirmed based on two separate semen analysis. Inclusion criteria for NOA were, having no history of genital infections and existence of bilateral vas deferens and the exclusion criteria were, having history of surgery or vasectomy. All OA cases were selected among men with primary idiopathic epididymis obstruction. Excluding criteria for OA cases were azoospermia due vas deferens or ejaculatory duct. Moreover, patients with genital infections, vasectomy, or other iatrogenic injuries to the male reproductive tract were excluded from the study. 

**Genotyping**

**Figure 1:**
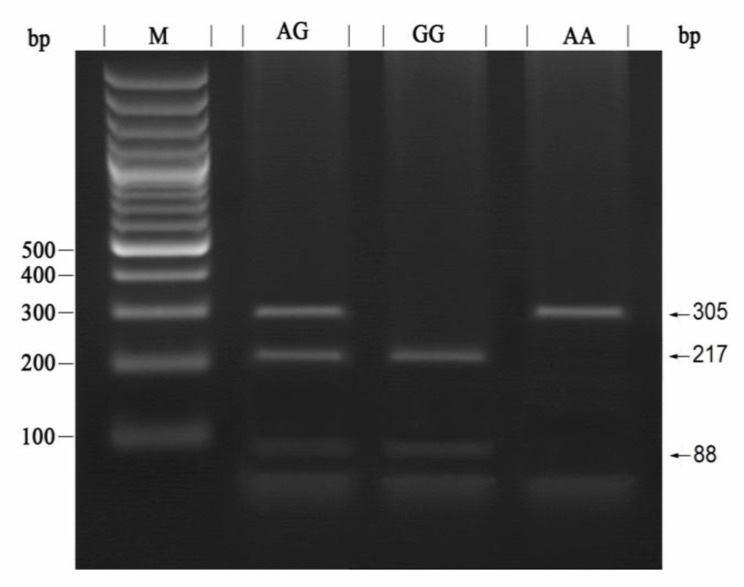
Identification of polymorphism at position A919G after digestion with AhdIrestriction enzyme.M: DNA Marker, AG: Heterozygous genotype digested to three fragments (305bp, 217bp, 88bp), GG: Homozygous for G allele digested to two fragments (217bp, 88bp), AA: Homozygous for A allele with no digestion

**Figure 2:**
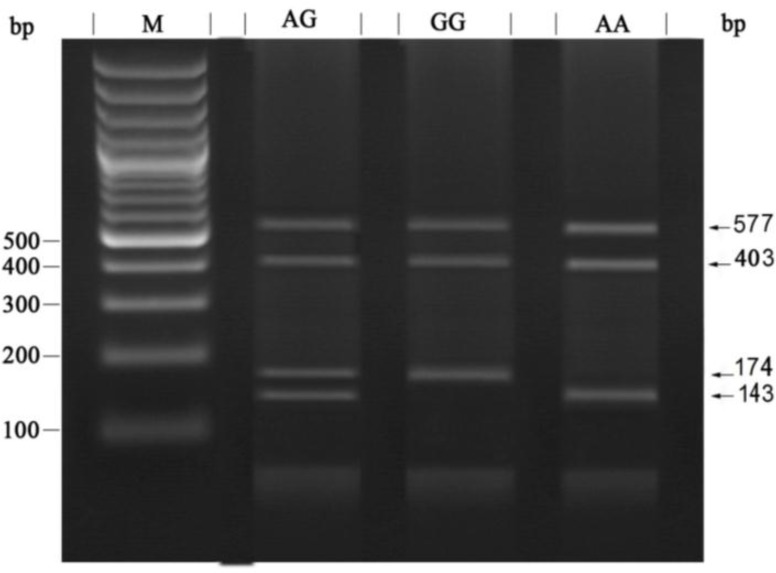
Identification of polymorphism at position A2039G after digestion with BsrI restriction enzyme.AG: Heterozygous genotype consisting of four fragments (577bp, 403bp, 174bp, 143bp). GG: Homozygous for G allele digested to three fragments (577bp, 403bp, 174bp). AA: Homozygous for A allele consisting of three fragments (57bp7, 403bp, 143bp). M: DNA Marker

DNA was extracted from the peripheral blood using PrimePrepTM Genomic DNA Isolation Kit according to the manufacturer’s protocol (Genet Bio, Korea). Locus specific primers were selected in order to amplify the FSHR gene polymorphisms at locus’s A919G and A2039Gby polymerase chain reaction-restriction fragment length polymorphism (PCR-RFLP) method. Primer sequences and PCR conditions are shown in Table I. For genotyping of the FSHR gene at position A919G, a 305bp product was amplified and subjected to digestion with AhdI (Fermentas, Lithuania) in order to create 217 and 88 bp fragments (Figure 1). The amplified PCR product for A2039G was 577 bp. After digestion with BsrI restriction enzyme (Fermentas, Lithuania), several bandssizing403, 174, and143 bp was created based on the genotypes. Finally, the digested bands were monitored by electrophoresis in 2.5% agarose gel (Figure 2). 

**FSH measurement**

Sera were separated from 2 ml of peripheral blood after centrifuging at 1000 x g for 10 minutes. All sera were stored at -70^°^C until assay. The level of FSH was measured using FSH ELISA kit (Vidas, BioMetrieux, France) according to the manufacturer's guideline and was compared to the results of genotyping of the FSHR.

**Statistical analysis**

Prior to the statistical analysis, Hardy-Weinberg equilibrium of expected and observed frequencies was evaluated using Alrequin 3.1 software algorithm. Subsequently, SPSS.16 (Statistical Package for the Social Sciences, version 16.0, SPSS Inc, Chicago, Illinois, USA) was used to perform the statistical analyses. Between-group comparisons were carried out using chi-square test. In order to determine which genotypes accounted for statistical differences, Bonferroni method was performed using the following formula p_c_=p×n, where the p_c_ is the corrected value, p is the uncorrected p-value, and n is the number of tests. P-value less than 0.05 was considered as statistically significant.

## Results

In this study, two SNPs of FSHR gene at positions A919G and A2039G were genotyped and the relationship with azoospermia was investigated in 212 azoospermic patients (126 non-obstructive and 86 obstructive) and 200 healthy Iranian men. The genotype frequencies in azoospermic patients and healthy controls were in concurrence with Hardy-Weinberg equilibrium. The results of the statistical analysis for A919G and A2039G genotypes and allele frequencies have presented in Tables II-V. 

Accordingly, AA genotype and A allele at A919G position were more frequent in OA cases in comparison with the healthy controls (P<0.001 for both comparisons, Tables II and III), although no significant difference was observed between NOA men and healthy controls regarding the distribution of genotypes and allele frequencies, AA genotypes and A allele were more frequent in OA compared to NOA patients (p<0.001 for both comparisons, Table II and Table III).

Considering A2039G polymorphism, no significant difference was found between OA and NOA patients as well as between the patients and the healthy controls regarding both genotype and allele frequencies (Table IV and Table V).

The findings of the current study indicated that the mean level of serum FSH was higher in the NOA men compared to the OA patients (23.8 versus 13.8, respectively, p=0.04). Additionally, analysis of the correlation between the serum FSH concentration and FSHR genotypes at positions A919G and A2039G indicated that OA patients with the AA genotype at position A919G had higher levels of FSH in comparison with those with genotypes GG and AG. However, no relationship was found between A2039G genotypes and FSH level in OA patients (P=0.04, Table VI). In addition, no significant association was observed between the levels of FSH and A919G or A2039G genotypes in NOA patients (Table VI).

**Table I:** Primers sequences and PCR conditions used for genotyping of FSHR SNPs

**SNP**	**Primer sequence**	**Product size**	**Annealing**
A919G	F 5´- CCT GCA CAA AGA CAG TGA TG -3´ R 5´- TGG CAA AGA CAG TGA AAA AG -3´	305 bp	58˚c
A2039G	F 5´- CAA AGA TTC TGC TGG TTC TG -3´ R 5´- ATC ATT CAA TAC TCA GAT ACA TT -3	577bp	52˚c

PCR: Polymerase chain reactio, FSHR: Follicle stimulating hormone, SNPs: Single nucleotide polymorphisms

**Table II:** Distribution of FSHR A919G genotypes frequencies in cases and healthy controls

**FSHR A919G Genotypes**	**OA number (%)**	**NOA number (%)**	**Healthy control number (%)**	**p-value**
AA	38 (44.2)	26 (20.6)	47 (23.5)	0.001*
GG	17 (19.8)	40 (31.8)	68 (34)	0.001**
AG	31 (36)	60 (47.6)	85 (42.5)	0.70***
Total	86 (100)	126 (100)	200 (100)	0.16****

OA: Obstructive azoospermia, NOA: Non-obstructive azoospermia

* OA versus controls, x^2^= 13.38,

** OA versus NOA, x^2^= 13.71,

*** NOA versus controls,

**** Total cases (OA and NOA) versus controls (Chi-Square test)

**Table III:** Distribution of FSHR A919G allele frequencies in cases and healthy controls

**FSHR A919G Alleles**	**OA** **n (%)**	**NOA** **n (%)**	**Healthy control** **n (%)**	**p-value**
A	107 (62.2)	112(44.4)	179 (44.7)	<0.001*
G	65 (37.8)	140 (55.6)	221 (55.3)	<0.001**
Total	172 (100)	252 (100)	400 (100)	0.06***

OA: Obstructive azoospermia, NOA: Non-obstructive azoospermia

* OA versus controls, X^2^= 13.98, OR= 2.03 (1.39< OR< 2.98), RR=1.65 (1.27< RR< 2.14),

** OA versus NOA, X^2^= 12.22, OR= 2.06 (1.36< OR< 3.12), RR= 1.54 (1.21< RR< 1.96),

*** Total cases (OA and NOA) versus controls, X^2^= 3.65, OR= 1.32 (0.99< OR< 1.75), RR= 1.14 (1.00< RR< 1.31), All p-values are calculated using Chi-Square test.

**Table IV:** Distribution of FSHR A2039G genotypes frequencies in cases and controls

**FSHR A2039G Genotypes**	**OA** **n (%)**	**NOA** **n (%)**	**Healthy control** **n (%)**	**p-value**
AA	16 (18.6)	17 (13.5)	40 (20)	0.61*
GG	21 (24.4)	30 (23.8)	53 (26.5)	0.10**
AG	49 (57)	79 (62.7)	107 (53.5)	0.33***
Total	86 (100)	126 (100)	200 (100)	0.14****

OA: Obstructive azoospermia, NOA: Non-obstructive azoospermia

* OA versus controls,

** NOA versus controls,

*** OA versus NOA,

**** Total cases (OA and NOA) versus controls (Chi-Square test)

**Table V:** Distribution of FSHR A2039G allele frequencies in cases and controls

**FSHR A2039G Genotypes**	**OA** **n (%)**	**NOA** **n (%)**	**Healthy control** **n (%)**	**p-value**
A	81 (47.1)	113 (44.8)	187 (47.7)	0.98*
G	91 (52.9)	139 (55.2)	213 (53.3)	0.72**
Total	172 (100)	252 (100)	400 (100)	0.82***

OA: Obstructive azoospermia, NOA: Non-obstructive azoospermia, N.S= Not significant

* OA versus controls,

** OA versus NOA,

*** Total cases (OA and NOA) versus controls (Chi-Square test)

**Table VI:** Genotype frequencies and serum FSH concentrations in obstructive and non-obstructive azoospermia

**FSHR SNP**	**Genotype**	**OA(MIU/ml) ** **(Mean ** **±** ** SD)**	**NOA (MIU/ml)** ** (Mean ** **±** ** SD)**	**p-value**
**A919G**	AA	21.7±18.5	34.4±34.2	<0.04*
	GG	3±4.8	21.4±18.1	
	AG	5.1±15.3	13.7±13.1	
**A2039G**	AA	9.3±12.9	30.1±25.7	0.20
	GG	33.1±17.9	6.1±10.8	
	AG	20.1±18.5	23.9±19.9	

OA: Obstructive azoospermia, NOA: Non-obstructive azoospermia, MIU= Milli-international unit, P-values are calculated using Student’s *t* test.

* AA versus GG genotype for OA patients, P= 0.04

## Discussion

Up to now, controversial findings have been obtained concerning the effect of SNPs within a FSHR gene on male infertility. Tüttelmann *et al*. conducted a meta-analysis in 2007 and did not find any association between male infertility and FSHR polymorphism at position A2039G (9). Nonetheless, they reported that A2039G polymorphisms in the FSHR gene might affect male reproductive parameter (10). Lend *et al*. in a meta-analysis published in 2010 reported that the G-29-A919-A2039 haplotype might be a protective factor against male sterility (11). On the other hand, Wu and colleagues in another meta-analysis in 2012 concluded that this haplotype might not be capable of causing susceptibility to male infertility (12). Furthermore, Tuerlings *et al*. suggested that mutations in the FSH receptor gene were not a common cause of infertility in azoospermic patients (13). In line with the study by Tuerlings *et al*., several previous reports indicated that FSHR SNPs at positions A919G and A2039G did not play any roles in susceptibility to spermatogenetic impairment, FSH concentration, sperm abnormalities, and azoospermia (14-17). In contrast to these studies, several researches have indicated a correlation between FSHR polymorphisms and azoospermia or infertility (7, 11, 18). The controversy in the previously published studies regarding the relationship between FSHR polymorphisms and fertility showed that several factors, such as ethnicity or the cause of infertility, might have affected the results. In order to resolve the contradiction in the previous studies, we conducted an experiment on 212 azoospermic cases and 200 normal controls who were age-and ethnic- matched and from the same geographic region (Fars province, southwest of Iran). Therefore, the genetic background, ethnicity, and environmental factors were also taken into account in the present study. Theoretically, FSH is an essential hormone for initiation of spermatogenesis at puberty and maintenance of quantitatively normal sperm production in humans. Interestingly, elevated FSH level is associated with spermatogenesis failure (19). FSH acts through the FSHRwhich is expressed only in Sertoli cells in humans. Various SNPs have been reported within the FSHR gene that may affect the receptor function. Both A919G and A2039G SNPs are important for glycosylation/phosphorylation of the receptor and play a role in the FSHR trafficking and turnover (20). Therefore, the level of FSH or genetic variant of its receptor would be expected to affect sperm production in males (12). Similar to the role of the FSHR SNPs in FSH plasma level, the relationship between FSHR A2039G polymorphism and FSH concentration has been reported previously (21). The results of the present study indicated that the presence of A allele at position 919 within FSHR gene might affect the receptor function and the FSH level. Both OA and NOA patients with the AA genotype at position 919 from FSHR had higher FSH plasma levels compared to those with AG or GG genotypes. Moreover, the level of FSH was elevated in the OA patients with the AA genotype at FSHR A919G position.

FSHR in interaction with its cognate receptors on Sertoli cells plays an important role in the maintenance of spermatogenesis. Moreover, FSHR polymorphisms, may affect receptor function and the levels of FSH. Mice knockout model for FSHR have fewer Sertoli cells and show abnormal and fewer germ cells compared to the normal wild type mice (22). 

In FSHR knockout mice, reduced fertility along with the lower levels of testosterone and androgen binding protein have been reported that might reflect changes to the epididymis (23). In primates, restricted FSHR signalling by the infant Sertoli cells may cause azoospermia (24). Lower levels of FSH, along with low levels of testosterones are indicative of pretesticular problems. Sperm parameters including sperm count, motility, and morphology all have been reported to significantly reduce in Fshr-/- mice (25). 

While multi unknown factors might increase susceptibility to OA, the results of the present study indicated that single nucleotide polymorphisms in FSHR gene, might account as one of the susceptible factors for the etiology of idiopathic epididymis is obstruction.

In conclusion, the present study findings demonstrated that the genetic polymorphisms in the FSHR gene might increase the susceptibility to OA in Iranian men. Moreover, genetic polymorphisms within the FSHR gene might affect FSH plasma level and spermatogenesis. Yet, further investigations are recommended to be conducted on other ethnic populations to confirm the results of this study.

## References

[1] Rostami Dovom M, Ramezani Tehrani F, Abedini M, Amirshekari G, Hashemi S, Noroozzadeh M (2014). A population-based study on infertility and its influencing factors in four selected provinces in Iran (2008-2010). Iran J Reprod Med.

[2] Esteves SC, Miyaoka R, Agarwal A (2011). An update on the clinical assessment of the infertile male. Clinics.

[3] Cavallini G, Beretta G, Biagiotti G (2011). Preliminary study of letrozole use for improving spermatogenesis in non-obstructive azoospermia patients with normal serum FSH. Asian J Androl.

[4] Esteves SC, Agarwal A (2013). Reproductive outcomes, including neonatal data, following sperm injection in men with obstructive and nonobstructive azoospermia: case series and systematic review. Clinics.

[5] Tapanainen JS, Aittomaki K, Min J, Vaskivuo T, Huhtaniemi IT (1997). Men homozygous for an inactivating mutation of the follicle-stimulating hormone (FSH) receptor gene present variable suppression of spermatogenesis and fertility. Nat Genet.

[6] Themmen AP (2005). An update of the pathophysiology of human gonadotrophin subunit and receptor gene mutations and polymorphisms. Reproduction.

[7] Ahda Y, Gromoll J, Wunsch A, Asatiani K, Zitzmann M, Nieschlag E (2005). Follicle-stimulating hormone receptor gene haplotype distribution in normozoospermic and azoospermic men. J Androl.

[8] Balkan M, Gedik A, Akkoc H, Ay O, Erdal ME, Isi H (2010). FSHR Single Nucleotide Polymorphism Frequencies in Proven Fathers and Infertile Men in Southeast Turkey. J Biomed Biotechnol.

[9] Tüttelmann F, Rajpert-De Meyts E, Nieschlag E, Simoni M (2007). Gene polymorphisms and male infertility--a meta-analysis and literature review. Reprod Biomed Online.

[10] Tüttelmann F, Laan M, Grigorova M, Punab M, Sõber S, Gromoll J (2012). Combined effects of the variants FSHB -211G>T andFSHR2039A>G on male reproductive parameters. J Clin Endocrinol Metab.

[11] Lend AK, Belousova A, Haller-Kikkatalo K, Punab M, Poolamets O, Peters M (2010). Follicle-stimulating hormone receptor genehaplotypes and male infertility in estonian population and meta-analysis. Syst Biol Reprod Med.

[12] Wu W, Cai H, Sun H, Lu J, Zhao D, Qin Y (2012). Follicle stimulating hormone receptor G-29A, 919A>G, 2039A>G polymorphism and the risk of male infertility: a meta-analysis. Gene.

[13] Tuerlings JH, Ligtenberg MJ, Kremer JA, Siers M, Meuleman EJ, Braat DD (1998). Screening male intracytoplasmic sperm injectioncandidates for mutations of the follicle stimulatinghormone receptor gene. Hum Reprod.

[14] Pengo M, Ferlin A, Arredi B, Ganz F, Selice R, Garolla A (2006). FSH receptor gene polymorphisms in fertile and infertile Italian men. Reprod Biomed Online.

[15] Ghirelli-Filho M, Peluso C, Christofolini DM, Gava MM, Glina S, Barbosa CP (2012). Variants in follicle-stimulating hormone receptor gene in infertile Brazilian men and the correlation to FSH serum levels and sperm count. Reprod Sci.

[16] Li JW, Gu YQ (2008). Predictors for partial suppression of spermatogenesis of hormonal male contraception. Asian J Androl.

[17] Ishikawa T, Fujisawa M, Tapanainen J (2006). Screening ofFSH receptorgene mutation (C566T) in azoospermic men in Japan. Arch Androl.

[18] Sheikhha MH, Eftekhar M, Kalantar SM (2011). Investigating the association between polymorphism of follicle-stimulating hormone receptor gene and ovarian response in controlled ovarian hyperstimulation. J Hum Reprod Sci.

[19] Bergmann M, Behre HM, Nieschlag E (1994). Serum FSH and testicular morphology in male infertility. Clin Endocrinol.

[20] Davis D, Liu X, Segaloff DL (1995). Identification of the sites of N-linked glycosylation on the follicle-stimulating hormone (FSH) receptor and assessment of their role in FSH receptor function. Mol Endocrinol.

[21] Morón FJ, Ruiz A (2010). Pharmacogenetics of controlled ovarian hyperstimulation: time to corroborate the clinical utility of FSH receptor genetic markers. Pharmacogenomics.

[22] Grover A, Sairam MR, Smith CE, Hermo L (2004). Structural and functional modifications of sertoli cells in the testis of adult follicle-stimulating hormone receptor knockout mice. BiolReprod.

[23] Grover A, Smith CE, Gregory M, Cyr DG, Sairam MR, Hermo L (2005). Effects of FSH receptor deletion on epididymal tubules and sperm morphology, numbers, and motility. MolReprod Dev.

[24] Majumdar SS, Sarda K, Bhattacharya I, Plant TM (2012). Insufficient androgen and FSH signalling may be responsible for the azoospermia of the infantile primate testes despite exposure to an adult-like hormonal milieu. Hum Reprod.

[25] Dierich A, Ram Sairam M, Monaco L, Maria Fimia G, Gansmuller A, LeMeur M (1998). Impairingfolliclestimulating hormone (FSH) signaling in vivo: targeted disruption of the FSH receptor leads to aberrant gametogenesis and hormonal imbalance. Proc Natl Acad Sci USA.

